# Correlations among the plasma concentrations of first-line anti-tuberculosis drugs and the physiological parameters influencing concentrations

**DOI:** 10.3389/fphar.2023.1248331

**Published:** 2023-10-06

**Authors:** Lin Cheng, Ming Luo, Yan Guo, Yunfan Fan, Pengsen Wang, Gang Zhou, Shiwei Qin, Bangbi Weng, Peibo Li, Zhirui Liu, Songtao Liu

**Affiliations:** ^1^ Department of Pharmacy, the First Affiliated Hospital of Army Medical University (Third Military Medical University), Chongqing, China; ^2^ Chongqing Public Health Medical Center, Southwest University Public Health Hospital, Chongqing, China; ^3^ Department of Infectious Diseases, the First Affiliated Hospital of Army Medical University (Third Military Medical University), Chongqing, China

**Keywords:** tuberculosis, isoniazid, rifampicin, ethambutol, pyrazinamide, drug concentration

## Abstract

**Background:** The plasma concentrations of the four most commonly used first-line anti-tuberculosis (TB) drugs, isoniazid (INH), rifampicin (RMP), ethambutol (EMB), and pyrazinamide (PZA), are often not within the therapeutic range. Insufficient drug exposure could lead to drug resistance and treatment failure, while excessive drug levels may lead to adverse reactions. The purpose of this study was to identify the physiological parameters influencing anti-TB drug concentrations.

**Methods:** A retrospective cohort study was conducted. The 2-h plasma concentrations of the four drugs were measured by using the high-performance liquid chromatography-tandem mass spectrometry method.

**Results:** A total of 317 patients were included in the study. The proportions of patients with INH, RMP, EMB, and PZA concentrations within the therapeutic range were 24.3%, 31.5%, 27.8%, and 18.6%, respectively. There were positive associations between the concentrations of INH and PZA and RMP and EMB, but negative associations were observed between the concentrations of INH and RMP, INH and EMB, RMP and PZA, and EMB and PZA. In the multivariate analysis, the influencing factors of the INH concentration were the PZA concentration, total bile acid (TBA), serum potassium, dose, direct bilirubin, prealbumin (PA), and albumin; those of the RMP concentration were PZA and EMB concentrations, weight, α-l-fucosidase (AFU), drinking, and dose; those of the EMB concentration were the RMP and PZA concentrations, creatinine, TBA and indirect bilirubin; and those of the PZA concentration were INH, RMP and EMB concentrations, sex, weight, uric acid and drinking.

**Conclusion:** The complex correlations between the concentrations of the four first-line anti-TB drugs lead to a major challenge in dose adjustment to maintain all drugs within the therapeutic window. Levels of TBA, PA, AFU, and serum potassium should also be considered when adjusting the dose of the four drugs.

## Introduction

`Tuberculosis (TB) is a major global public health problem, and China is one of the countries with a heavy burden of TB (Chakaya et al., 2021). Drug therapy is an important method for *tuberculosis* prevention and control, and the combined application of multiple drugs is characteristic of anti-TB therapy, which has a long-term therapeutic course and is associated with many adverse reactions. Currently, the recommended dose of anti-TB drugs is based on the body mass of patients. Due to individual differences in pharmacokinetics, drug concentrations vary widely among patients (Kumar et al., 2018). Insufficient drug exposure could lead to drug resistance and treatment failure (Weiner et al., 2003; Dheda et al., 2018), while excessive drug levels may lead to adverse reactions (Martson et al., 2021). Therefore, therapeutic drug monitoring (TDM) is recommended for anti-TB drugs (Alsultan and Peloquin, 2014). Isoniazid (INH), rifampicin (RMP), ethambutol (EMB), and pyrazinamide (PZA) are the most commonly used first-line anti-TB drugs in clinical practice (Furin et al., 2019). Previous studies showed that low blood concentrations of INH and RMP were present in more than 70% of patients treated with these drugs (Park et al., 2016; Stott et al., 2018). The month 2 culture conversion TB patients had lower concentrations of INH, EMB, and PZA than the month 1 TB patients (Lei et al., 2019). Therefore, it is urgent to investigate the factors influencing the plasma concentrations of first-line anti-TB drugs (Prahl et al., 2014; Park et al., 2016) to optimize individualized dosage regimens, improve drug efficacy, and reduce related adverse reactions.

Evidence from a previous study suggested that INH, RMP, EMB, and PZA all reach peak serum concentrations approximately 2 h after administration (Alsultan and Peloquin, 2014). In Chinese patients with TB, the recommended therapeutic windows of the four drugs are defined as follows: INH, 3–6 μg/ml; RMP, 8–24 μg/ml; PZA, 20–60 μg/ml; and EMB, 2–6 μg/ml (Lu and Zhu, 2021). Therefore, investigating the influencing factors of the serum concentration of the four drugs at 2 h after administration may provide a reference for the rational use of the drugs (Sharma et al., 2018; Zhao et al., 2022). Patients’ baseline characteristics, drug administration route and dose, blood uric acid (UA) level, serum albumin level, serum creatinine level, total bilirubin (TBIL) level, and blood urea nitrogen level have been identified as influencing factors of the four anti-TB drug plasma concentrations (Zhao et al., 2022). However, they did not consider the drug‒drug interactions (DDIs) among the four drugs, and the predictive value of indicators was not determined.

INH is mainly acetylated to acetyl isoniazid and isoniazid acid by N-acetyltransferase 2 in the liver (Metushi et al., 2016). INH can elevate plasma concentrations of companion drugs mainly by inhibiting CYP3A4 or 2C19 (Peloquin and Davies, 2021). RMP is also involved in numerous DDIs since it is a strong inducer of multiple CYP isoforms, including CYP3A4, 2A6, 2B6, 2C9 and 2C19, and the majority of prescription drugs are metabolized by one or more of these isoforms (Niemi et al., 2003; Nahid et al., 2016). PZA is mainly metabolized in the liver by microsomal dehydrogenase and xanthine oxidase, hydrolyzed into pyrazinoic acid, and then hydroxylated into inactive metabolites, which are excreted by glomerular filtration (Stemkens et al., 2019). Most EMB is excreted in its original form through the kidney, and kidney function has a certain effect on the EMB plasma concentration (Denti et al., 2015). Therefore, impaired liver function and renal function may influence the concentrations of the four drugs, and there may be some correlations of drug exposure among the four drugs.

The objectives of this study were to investigate the correlations of 2-h plasma concentrations among the four first-line anti-TB drugs, identify physiological parameters influencing the plasma concentrations of the four drugs through a stepwise multivariable linear regression model, and estimate the predictive value of indicators by using receiver operating characteristic (ROC) curve analysis.

## Materials and methods

### Ethics statement

The study was conducted in accordance with the Declaration of Helsinki, and the protocol of the study was approved by the Ethics Committee of Chongqing Public Health Medical Center (2023–005-02-KY). All methods were carried out in accordance with relevant guidelines and regulations. The Ethics Committee of Chongqing Public Health Medical Center approved this study to be exempt from individual patient consent for publication as existing data were collected and de-identified.

### Patients and study design

Adult patients with TB hospitalized in Chongqing Public Health Medical Center from September 2021 to July 2022, undergoing 2-h plasma concentration measurements of INH, RMP, EMB, and PZA, were included in the current retrospective study. Patients receiving the treatment for the first time or patients on chronic treatment were both included. Patients who were coinfected with human immunodeficiency virus or who had diseases or symptoms that affect drug metabolism were excluded.

### Data collection

The following demographic and medical details, several indicators of liver function and renal function and electrolytes within 3 days of drug concentration measurement of each eligible patient, were collected: a) sex, age, weight, underlying diseases, drinking history, and drug dose; b) prealbumin (PA), α-l-fucosidase (AFU), glutamic-pyruvic transaminase (ALT), glutamic-oxaloacetic transaminase (AST), AST/ALT, γ-glutamyl transferase (γ-GT), alkaline phosphatase (ALP), total bile acid (TBA), TBIL, direct bilirubin (DBIL), indirect bilirubin (IBIL), albumin, and cholinesterase (CHE); c) serum urea and creatinine levels, UA, cystatin C (CysC) and estimated glomerular filtration rate (eGFR); and d) serum potassium, sodium and magnesium.

### Measurement of drug concentrations

Venous blood was drawn 2 h after drug ingestion under fasting conditions. Plasma was separated after centrifugation at 3,500 rpm for 10 min at 4 °C. An aliquot of 100 μL of plasma was placed into tubes containing 400 μL of 50% methanol. After 2 min of vortexing, the mixtures were centrifuged at 4 °C and 15,000 rpm for 10 min. The supernatant (20 μL) was transferred into a new tube containing 180 µL of 100% methanol, and these mixtures were then centrifuged at 4 °C and 15,000 rpm for 3 min. The supernatant (2 μL) was injected into the LC‒MS/MS system for analysis (Song et al., 2007). The method was validated over the concentration range of 0.25–50 μg/ml for INH, RMP, and EMB and 1.0–200 μg/ml for PZA. The lower limit of quantification was 0.25 μg/ml for INH, RMP, and EMB and 1.0 μg/ml for PZA.

### Statistical analysis

The statistical analysis was performed by using IBM SPSS 19.0 (IBM Corp., Armonk, NY, United States). The correlations among drug concentrations and indicators were evaluated by Pearson’s correlation analysis. To investigate the independent influencing factors of plasma concentrations of INH, RMP, EMB, and PZA, 25 factors, including sex, age, dose, drinking history, and biochemical indicators, were included in the stepwise multivariable linear regression model. The predictive effect of UA and TBA for the concentrations of the four drugs was determined by receiver operating characteristic (ROC) curve analysis. A *p*-value of <0.05 was considered statistically significant.

## Results

### Patient characteristics

A total of 317 samples from 317 patients were included in the current study. The percentage of treatment-naïve patients was 55.5% (176/317). The demographic and clinical data of the patients are shown in Table 1 and Table 2. The proportion of males was 68.5%, with ages of 18–91 years and weights of 31–105 kg, and 50.8% of patients did not have a drinking history. The primary administered doses of first-line drugs were as follows: INH, 300 mg/d; RMP, 450 mg/d; EMB, 750 mg/d; and PZA, 1,500 mg/d. The proportions of patients with INH, RMP, EMB, and PZA concentrations within the therapeutic range were 24.3%, 31.5%, 27.8%, and 18.6%, respectively.

**TABLE 1:** Demographic and clinical characteristics of the included patients.

Characteristics	Included patients (n = 317)
Sex	
Male (n [%])	217 (68.5)
Female (n [%])	100 (31.5)
Age (years)	47 ± 17
BMI	20.8 ± 3.6
Drinking	
No (n [%])	161 (50.8)
Previous (n [%])	86 (27.1)
Continue (n [%])	61 (19.2)
Unknown (n [%])	9 (2.8)
Drug dosage	
INH (mg/d)	
150	1 (0.3)
200	1 (0.3)
300	286 (90.2)
400	12 (3.8)
500	5 (1.6)
600	12 (3.8)
RMP (mg/d)	
300	3 (0.9)
450	216 (68.1)
600	98 (30.9)
EMB (mg/d)	
375	1 (0.3)
500	2 (0.6)
750	306 (96.5)
1,000	8 (2.5)
PZA (mg/d)	
750	1 (0.3)
1,000	6 (1.9)
1,500	308 (97.2)
2000	1 (0.3)
2,250	1 (0.3)
2-h plasma concentration	
INH (μg/ml)	
<3	211 (66.6)
3–6	77 (24.3)
>6	29 (9.1)
RMP (μg/ml)	
<8	56 (17.7)
8–24	100 (31.5)
>24	161 (50.8)
EMB (μg/ml)	
<2	77 (24.3)
2–6	88 (27.8)
>6	152 (47.9)
PZA (μg/ml)	
<20	245 (77.3)
20–60	59 (18.6)
>60	13 (4.1)

Abbreviations: BMI, body mass index; INH, isoniazid; RMP, rifampicin; EMB, ethambutol; PZA, pyrazinamide.

**TABLE 2:** Biochemical indicators of the included patients.

Characteristics	Range	Mean ± SD or median (IQR)
Drug dosage		
INH (mg/d)	150–600	317 ± 64
RMP (mg/d)	300–600	495 ± 72
EMB (mg/d)	375–1,000	754 ± 50
PZA (mg/d)	750–2,250	1,491 ± 98
Biochemical indicators		
PA (100–400 mg/L)	39–444	193 ± 68
AFU (3–40 U/L)	6.91–63.70	26.20 ± 9.55
ALT (9–50 U/L)	2–289	13 (9, 19)
AST (15–40 U/L)	5–1,149	19 (16, 24)
AST/ALT (0–1.5)	0.5–5.8	1.67 ± 0.79
ALP (45–125 U/L)	11–420	91.0 ± 36.4
γ-GT (10–60 U/L)	9–591	22 (16, 44)
TBIL (1.71–26 μmol/L)	3.3–43.1	13.7 ± 6.4
DBIL (0–8.6 μmol/L)	0.1–21.9	4.9 ± 3.1
IBIL (0–16 μmol/L)	0.9–24.0	8.8 ± 4.0
TBA (0–15 μmol/L)	0.7–149.9	4.3 (2.6, 7.3)
Albumin (40–55 g/L)	24.2–47.4	37.6 ± 4.5
CHE (4,000–13000 kU/L)	1848–15859	7,346 ± 2,194
Urea (2.2–8.3 mmol/L)	1.53–27.15	4.47 (3.56, 5.52)
Creatinine (40–106 μmol/L)	28.3–579.7	60.9 ± 34.4
UA (210–430 μmol/L)	83–981	452.4 ± 182.2
Cystatin C (0.54–1.3 mg/L)	0.55–7.82	1.14 ± 0.51
eGFR (77–200)	9–179	112.8 ± 19.7
Serum potassium (3.5–5.3 mmol/L)	2.86–5.36	4.02 ± 0.41
Serum sodium (135–145 mmol/L)	125.8–147.7	139.8 ± 3.4
Serum magnesium (0.75–1.08 mmol/L)	0.60–1.06	0.82 ± 0.07
2-h plasma concentration		
INH (μg/ml)	0.33–17.40	2.15 (1.39, 3.66)
RMP (μg/ml)	0.40–91.64	26.68 (12.04, 42.74)
EMB (μg/ml)	0.26–21.93	5.89 (2.18, 9.73)
PZA (μg/ml)	0.39–100.98	4.52 (2.80, 15.74)

Abbreviations: INH, isoniazid; RMP, rifampicin; EMB, ethambutol; PZA, pyrazinamide; PA, prealbumin; AFU, α-l-fucosidase; ALT, glutamic-pyruvic transaminase; AST, glutamic-oxalacetic transaminase; ALP, alkaline phosphatase; γ-GT, γ-glutamyl transferase; TBIL, total bilirubin; DBIL, direct bilirubin; IBIL, indirect bilirubin; TBA, total bile acid; UA, uric acid; Cysc, cystatin C; eGFR, glomerular filtration rate; CHE, cholinesterase.

### Correlations between drug concentrations and indicators

**FIGURE 1:**
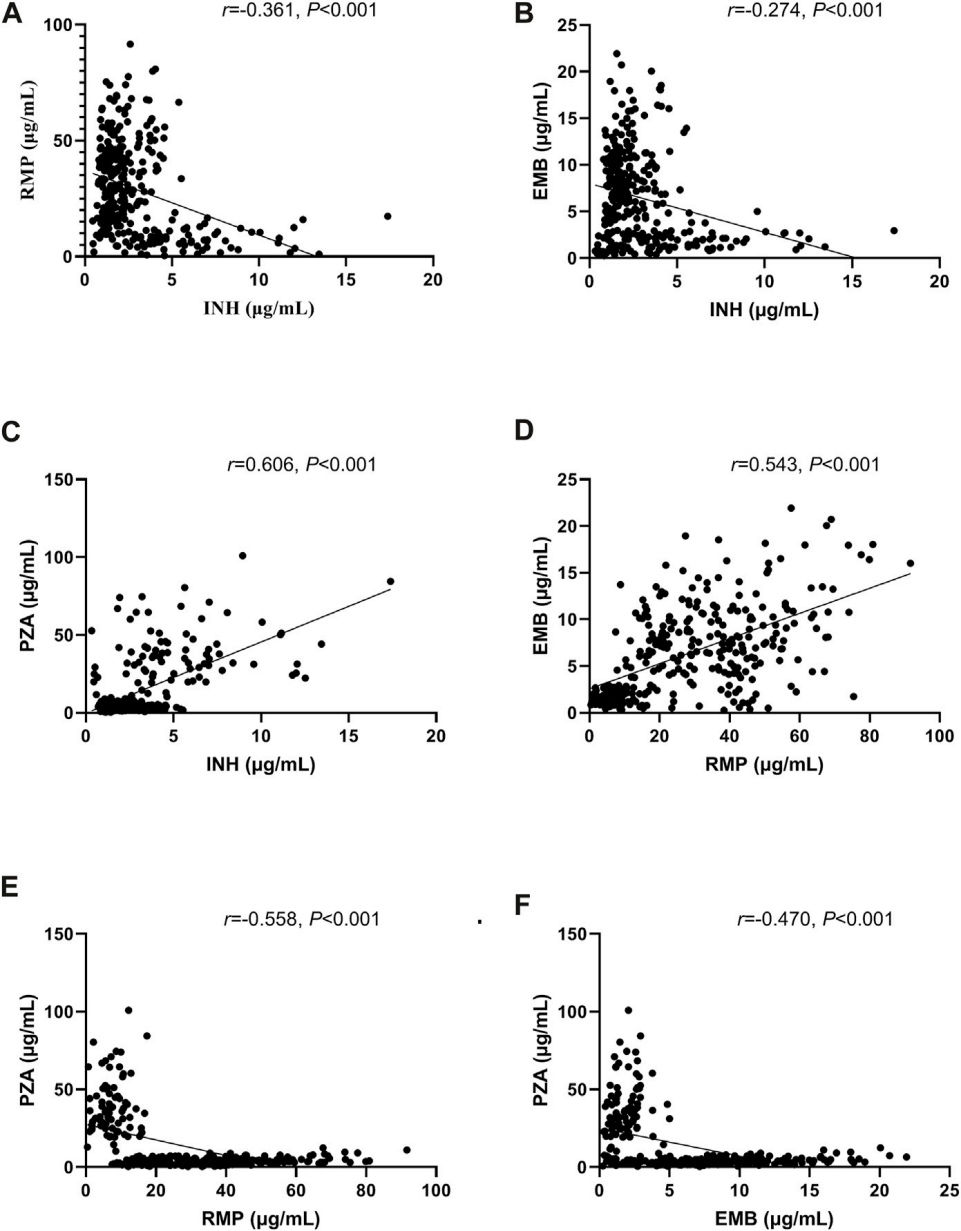
Correlations of concentrations between the first-line anti-tuberculosis (TB) drugs isoniazid (INH) and rifampicin (RMP) **(A)**, INH and ethambutol (EMB) **(B)**, INH and pyrazinamide (PZA) **(C)**, RMP and EMB **(D)**, RMP and PZA **(E)**, and EMB and PZA **(F)**.

The correlations between variables and the plasma concentration of first-line anti-TB drugs are shown in Table 3. Except for albumin, γ-GT and eGFR, other indicators showed correlations with the concentration of at least one of the drugs. Levels of UA were positively associated with concentrations of INH and PZA (r = 0.230, *p* < 0.001; r = 0.343, *p* < 0.001) but negatively associated with concentrations of RMP and EMB (r = -0.332, *p* < 0.001; r = -0.258, *p* < 0.001). The concentration of INH was negatively associated with the concentrations of RMP and EMB (r = -0.361, *p* < 0.001; r = -0.274, *p* < 0.001) but positively associated with the concentration of PZA (r = 0.606, *p* < 0.001); the concentration of RMP was positively associated with the concentration of EMB (r = 0.543, *p* < 0.001) but negatively associated with the concentration of PZA (r = -0.558, *p* < 0.001); and the concentration of EMB was negatively associated with the concentration of PZA (r = -0.470, *p* < 0.001) (Figure 1).

**TABLE 3:** Correlations between variables and the plasma concentration of first-line anti-tuberculosis drugs.

Variable	INH		RMP		EMB		PZA	
	*r*	*p-value*	*r*	*p-value*	*r*	*p-value*	*r*	*p-value*
Age (years)	0.055	0.191	-0.142	0.013	0.026	0.344	0.024	0.352
Weight (kg)	-0.155	0.007	-0.284	<0.001	-0.179	0.002	-0.112	0.039
Dose (mg)	0.130	0.019	-0.227	<0.001	-0.104	0.051	0.233	<0.001
PA (mg/L)	-0.198	0.001	0.003	0.481	-0.040	0.267	-0.141	0.013
AFU (U/L)	0.104	0.048	-0.273	<0.001	-0.051	0.212	0.157	0.006
ALT (U/L)	0.168	0.004	-0.072	0.128	0.004	0.474	0.081	0.100
AST (U/L)	0.233	<0.001	-0.070	0.136	-0.044	0.248	0.145	0.011
AST/ALT	0.203	0.001	0.023	0.359	-0.077	0.113	0.169	0.004
ALP (U/L)	0.094	0.068	-0.147	0.010	0.005	0.470	0.065	0.153
γ-GT (U/L)	0.060	0.172	-0.052	0.205	0.049	0.223	0.005	0.471
TBIL (μmol/L)	0.134	0.017	0.062	0.164	0.116	0.034	0.054	0.198
DBIL (μmol/L)	0.250	<0.001	-0.046	0.237	0.040	0.265	0.124	0.024
IBIL (μmol/L)	0.029	0.323	0.131	0.019	0.154	0.008	-0.005	0.467
TBA (μmol/L)	0.301	<0.001	-0.082	0.099	0.041	0.261	0.131	0.019
Albumin (g/L)	-0.054	0.355	0.002	0.968	0.004	0.945	-0.060	0.311
CHE (kU/L)	-0.106	0.046	-0.157	0.007	-0.060	0.173	-0.012	0.424
Urea (mmol/L)	0.162	0.005	-0.074	0.121	0.011	0.431	0.030	0.317
Creatinine (μmol/L)	0.056	0.188	-0.045	0.238	-0.128	0.023	-0.039	0.272
UA (μmol/L)	0.230	<0.001	-0.332	<0.001	-0.258	<0.001	0.343	<0.001
Cystatin C (mg/L)	0.142	0.012	-0.098	<0.001	-0.044	0.244	0.029	0.323
eGFR	-0.079	0.104	0.088	0.083	0.044	0.244	-0.007	0.457
Serum potassium (mmol/L)	0.139	0.013	-0.093	0.071	-0.046	0.237	0.064	0.155
Serum sodium (mmol/L)	-0.160	0.005	0.037	0.281	0.072	0.131	-0.101	0.055
Serum magnesium (mmol/L)	-0.074	0.119	0.143	0.012	0.181	0.002	-0.113	0.037

Abbreviations: INH, isoniazid; RMP, rifampicin; EMB, ethambutol; PZA, pyrazinamide; PA, prealbumin; AFU, α-l-fucosidase; ALT, glutamic-pyruvic transaminase; AST, glutamic-oxalacetic transaminase; ALP, alkaline phosphatase; γ-GT, γ-glutamyl transferase; TBIL, total bilirubin; DBIL, direct bilirubin; IBIL, indirect bilirubin; TBA, total bile acid; UA, uric acid; eGFR, estimated glomerular filtration rate; CHE, cholinesterase.

### Factors influencing drug concentrations

In the multivariate analysis, the PZA concentration; levels of TBA, DBIL, PA and albumin; dose; and serum potassium were the independent influencing factors of the INH concentration. PZA and EMB concentrations, weight, levels of UA, drinking and dose were the independent influencing factors of the RMP concentration. RMP and PZA concentrations, levels of creatinine, TBA and IBIL were the independent influencing factors of the EMB concentration. INH, RMP, and EMB concentrations, levels of UA, weight and drinking were the independent influencing factors of the PZA concentration (Table 4).

**TABLE 4:** Influencing factors of the plasma concentration of first-line anti-tuberculosis drugs.

INH			RMP			EMB			PZA		
Factor	OR (95% CI)	*p*-Value	Factor	OR (95% CI)	*p*-Value	Factor	Or (95% CI)	*p*-Value	Factor	Or (95% CI)	*p*-Value
Constant	-6.288 (-9.991, -2.584)	0.001	Constant	86.583 (70.267, 102.899)	<0.001	Constant	5.865 (3.486, 8.244)	<0.001	Constant	36.049 (19.321, 52.778)	<0.001
PZA concentration	0.068 (0.055, 0.080)	<0.001	PZA concentration	-0.483 (-0.588, -0.377)	<0.001	RMP concentration	0.090 (0.060, 0.120)	<0.001	INH concentration	2.763 (2.025, 3.501)	<0.001
TBA	0.048 (0.014, 0.082)	0.006	Weight	-0.508 (-0.697, -0.319)	<0.001	PZA concentration	-0.067 (-0.097, -0.037)	<0.001	PMP concentration	-0.368 (-0.476, -0.260)	<0.001
Serum potassium	0.960 (0.381, 1.539)	0.001	EMB concentration	1.044 (0.611, 1.476)	<0.001	Serum creatinine	-0.048 (-0.079, -0.017)	0.002	Sex	3.607 (-0.369, 7.582)	0.075
Dose	0.007 (0.003, 0.011)	0.001	AFU	-0.310 (-0.499, -0.121)	0.001	TBA	0.080 (0.020, 0.140)	0.010	EMB concentration	-0.790 (-1.196, -0.384)	<0.001
DBIL	0.102 (0.005, 0.199)	0.040	Drinking	-3.182 (-5.396, -0.968)	0.005	IBIL	0.136 (0.013, 0.258)	0.030	Weight	-0.345 (-0.526, -0.163)	<0.001
PA	0.007 (-0.012, -0.003)	0.003	Dose	-0.029 (-0.056, -0.003)	0.032				Drinking	-2.854 (-5.147, -0.562)	0.015
Albumin	0.078 (0.004, 0.153)	0.040							UA	0.011 (0.002, 0.021)	0.015

Abbreviations: INH, isoniazid; RMP, rifampicin; EMB, ethambutol; PZA, pyrazinamide; TBA, total bile acid; UA, uric acid; PA, prealbumin; DBIL, direct bilirubin; AFU, α-l-fucosidase; IBIL, indirect bilirubin.

### Exposure cutoff values for drug concentrations

**FIGURE 2:**
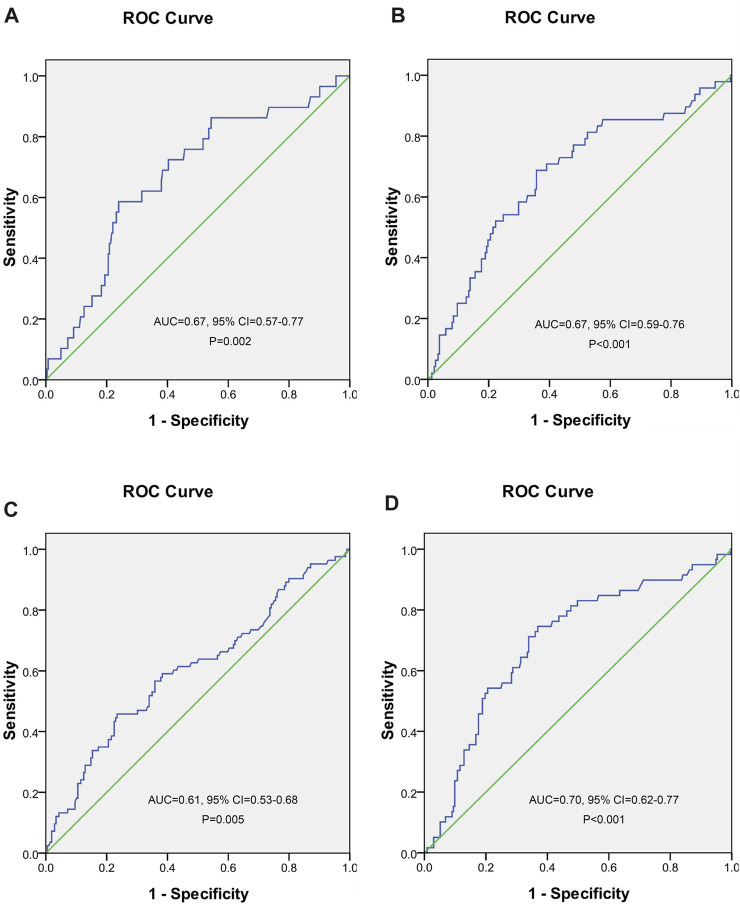
Receiver operating characteristic curve of blood uric acid for predicting concentrations of isoniazid **(A)**, rifampicin **(B)**, ethambutol **(C)**, and pyrazinamide **(D)**.

**FIGURE 3:**
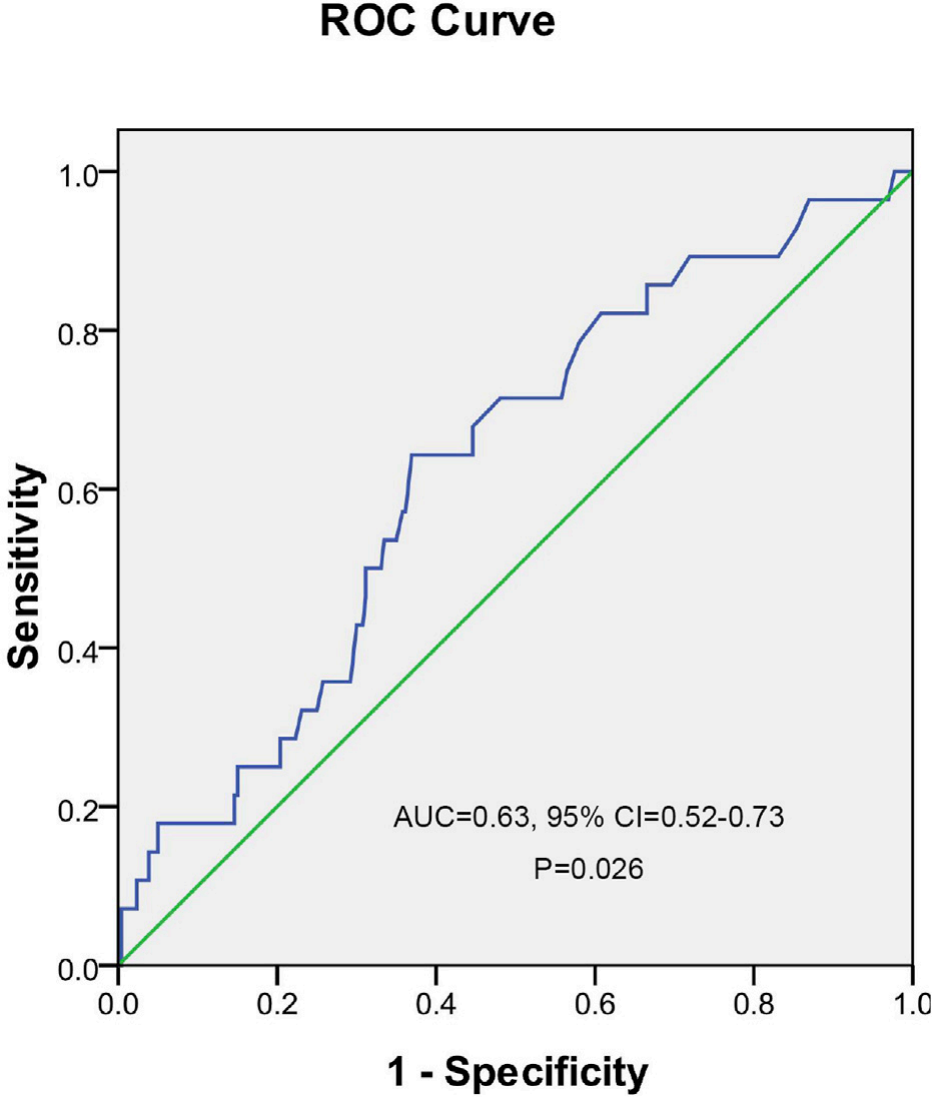
Receiver operating characteristic curve of total bile acid for predicting the concentration of isoniazid.

A ROC curve analysis was subsequently performed to calculate the cutoff points of drug concentrations. The cutoff value of UA with the largest Youden index for INH concentrations higher than 6 μg/ml was 562.5 μmol/L, the cutoff value of UA with the largest Youden index for RMP concentrations <8 μg/ml was 486.5 μmol/L, the cutoff value of UA with the largest Youden index for EMB concentrations between 2 and 6 μg/ml was 549.5 μmol/L, and the cutoff value of UA with the largest Youden index for PZA concentrations between 20 and 60 μg/ml was 462.5 μmol/L (Figure 2). The cutoff value of TBA with the largest Youden index for INH concentrations higher than 6 μg/ml was 5.05 μmol/L (Figure 3).

## Discussion

In the clinic, doctors often encounter the phenomenon of an inability of a drug to reach therapeutic blood concentration levels, even after adjusting to the maximum dose, but the reason is not clear. Currently, doctors are still using the traditional standard dosing method and consider changing the drug dose when resistance or adverse events appear. In the current work, a total of 317 patients were included, and the relationships of patient demographic characteristics and major biochemical indicators prior to drug administration with 2-h plasma concentrations of anti-TB drugs were analyzed by a stepwise multivariable linear regression model. The results showed that the proportion of patients with anti-TB drug concentrations in therapeutic ranges was very low: 66.6% of patients had lower INH concentrations, 50.8% of patients had higher RMP concentrations, 47.9% of patients had higher EMB concentrations, and 77.3% of patients had lower PZA concentrations than the therapeutic concentration ranges. Low serum INH may play a role in recurrence and in acquired drug resistance (Park et al., 2016). The microbiological efficacy of PZA increases with increasing drug concentrations; however, optimizing the PZA dose alone is unlikely to be sufficient to allow TB treatment shortening; rather, the RMP dose would need to be increased in parallel (Zhang et al., 2021). Based on our results, there was a negative correlation between the concentrations of PZA and RMP. The complex correlations will pose a severe challenge in the dose adjustment to maintain all drugs within the therapeutic window. INH is a weak inhibitor of CYP3A4 or 2C19, while RMP is a strong inducer of CYP3A4, 2A6, 2B6, 2C9 and 2C19, and most EMB is excreted in its original form through the kidney. Therefore, weak correlations were found between INH and RMP and INH and EMB in our work.

A large number of previous studies have shown that anti-TB drugs can affect UA metabolism in humans, which could result in elevated blood UA (Louthrenoo et al., 2015; Thumamo Pokam et al., 2018; Ha et al., 2019). We also found that UA was an independent influencing factor of the PZA concentration. However, the association of UA with anti-TB drug concentrations and the predictive value of UA for the concentrations were complex. When the concentration of UA was elevated, the possibilities of INH concentrations higher than 6 μg/ml and RMP concentrations below 8 μg/ml were increased, but EMB and PZA concentrations were prone to be maintained in the normal range. The mechanisms of the interaction between blood UA and anti-TB drug metabolism need to be further investigated.

Low-level INH may result in INH monoresistant and multidrug resistant (MDR) TB, ultimately affecting the treatment outcome (Nagel et al., 2017). In the current work, the proportion of patients receiving 300 mg INH was 90.2%, but 66.6% of patients had INH concentrations that were lower than the therapeutic range. Previous studies showed that drug dose was positively correlated with the plasma concentration of INH (Um et al., 2007; Zhao et al., 2022), which is consistent with the results of the present study, indicating that an insufficient dose is an important factor that leads to subtherapeutic concentrations. Data showed that 80% of Asian patients presented a fast-acetylation genotype of INH, leading to low exposure to INH, and higher INH doses should be used in fast acetylators (Peloquin and Davies, 2021). The patients included in our study were not genotyped for their acetylation status, but we suspected that the proportion of the fast-acetylation genotype of INH would be high. Using INH 900 mg, 3 times weekly, may be a choice for this patient group.

TBA can effectively reflect liver cell injury and the secretion and synthesis function of liver cells, and it is elevated before bilirubin increases. PA is synthesized mainly by liver cells and has a shorter half-life than albumin, which decreases before albumin decreases and can sensitively reflect the degree of early liver injury. In addition to UA, the liver function indicators TBA, PA, and DBIL were also found to be associated with the INH concentration. INH may induce hypokalemia (Molie et al., 2019). Our results also showed that serum potassium was significantly associated with the INH concentration. Therefore, except for previously well-known influencing factors, levels of TBA, PA, and serum potassium should also be considered when adjusting the dose of INH.

A positive correlation between RMP plasma concentrations and serum albumin levels has been reported (Tappero et al., 2005; Abulfathi et al., 2019; Lei et al., 2019), which may be attributed to the albumin binding rate of the drug. In our results, we did not find marked effects of albumin on the RMP concentration, possibly because most patients had lower albumin levels (65.8%). Weight showed a significant effect on the RMP concentration, which indicated that the dose of RMP should be adjusted individually. In the current work, 50.8% of patients had RMP concentrations higher than the therapeutic range. This phenomenon may be attributed to the fact that 31% of patients received 600 mg/d RMP. Previous studies showed that higher RMP doses were associated with more rapid sputum sterilization but with similar toxicity (Velasquez et al., 2018). The level of AFU may be elevated in approximately 40% of patients with hepatitis and 30% of patients with cirrhosis (Johnson, 2001). We also found that AFU was an independent influencing factor of the RMP concentration. The level of AFU should be considered when setting the dose of RMP.

Seventy percent of EMB is excreted unchanged in the urine, and elimination is closely related to renal function. In our results, serum creatinine, except for TBA or IBIL, was significantly associated with the EMB concentration, which indicated that serum creatinine should be considered when using EMB.

High exposure to PZA and its metabolites may result in hepatotoxicity, whereas low exposure to PZA has been correlated with the failure of first-line anti-TB therapy (Sundell et al., 2021). Our results showed that most patients’ PZA concentrations were below the therapeutic range (77.3%), which indicated that the patients were at risk of treatment failure. In the process of metabolizing alcohol, the metabolites, ethanol and metabolic derivatives produced by the liver will cause inflammation in the liver, and the inflammation will cause damage to the liver cells and even necrosis, leading to the occurrence of alcoholic hepatitis (Hyun et al., 2021). We found that drinking history was an independent influencing factor of RMP and PZA concentrations. The results highlight the importance of temperance in receiving first-line anti-TB drugs.

The following limitations should be noted. First, this was a single-center retrospective study, and the sample was relatively small. A multicenter, prospective, and large sample study is needed to confirm our results. Second, we analyzed the serum concentration only at 2 h after administration, which may lead to certain bias. Third, in the current study, the sensitivity and specificity observed for the cutoff values of UA and TBA were low. This finding was predictable because of the multiple influencing factors of the concentrations of each drug in the multivariable analysis. However, we could also obtain beneficial findings from the cutoff values to some extent. In addition, it is necessary to analyze the relationship between the drug concentration and drug efficacy and patient prognosis.

In conclusion, the correlations among the four first-line anti-TB drug concentrations were complex, which leads to a major challenge in dose adjustments to maintain all drugs within the therapeutic window. In addition to previously well-known influencing factors, including age, sex, weight, dose, UA, DBIL, and IBIL, the levels of TBA, PA, AFU, and serum potassium should also be considered when adjusting the dose of the four drugs. During long-term administration of the four first-line anti-TB drugs, plasma concentrations of the drugs and relevant indicators should be monitored to optimize the dosing schedule, and the concentration prediction model derived from our multivariate analysis may be referenced.

## Data Availability

The raw data supporting the conclusion of this article will be made available by the authors, without undue reservation.
